# Loss of FBXL14 promotes mesenchymal shift and radioresistance of non-small cell lung cancer by TWIST1 stabilization

**DOI:** 10.1038/s41392-021-00599-z

**Published:** 2021-07-21

**Authors:** Yan-Hong Cui, Jae-Hyeok Kang, Yongjoon Suh, Yi Zhao, Joo Mi Yi, In-Hwa Bae, Hae-June Lee, Dong Won Park, Min-Jung Kim, Su-Jae Lee

**Affiliations:** 1 Department of Life Science, Research Institute for Natural Sciences, Hanyang University, Seoul, Korea; 2 Department of Medicine, Section of Dermatology, University of Chicago, Chicago, IL USA; 3 Department of Microbiology and Immunology, Inje University, Busan, South Korea; 4 Division of Basic Radiation Bioscience, Korea Institute of Radiological & Medical Sciences, Seoul, Korea; 5 Division of Radiation Effect, Korea Institute of Radiological and Medical Sciences, Seoul, Korea; 6 Department of Internal Medicine, College of Medicine, Hanyang University, Seoul, Korea; 7 Laboratory of Radiation Exposure and Therapeutics, National Radiation Emergency Medical Center, Korea Institute of Radiological and Medical Sciences, Seoul, Korea

**Keywords:** Lung cancer, Molecular biology

**Dear Editor,**

Lung cancer is the most common cause of cancer-related mortality worldwide, with non-small cell lung cancer (NSCLC) accounting for the largest number of cases.^1^ Ionizing radiation (IR) is widely used as an indispensable tool for treating lung cancer patients; however, the acquisition of resistance following radiation is the major obstacle for reducing the efficacy of radiotherapy.^2^ To overcome this limitation, it is important to determine the mechanism by which cancer cells become resistant to radiation. Previously, E3 ubiquitin ligases were suggested as a potential target for radiosensitization in cancer therapy.^3^

To investigate the differences in the genetic profiles of radioresistant (RR) and radiosensitive (RS) patients, NSCLC samples (GSE42127 and GSE10072) from GEO datasets were classified into the RR/RS groups (Supplementary Fig. 1a, b), using the hierarchical clustering module of GenePattern based on 31 radiosensitivity signature genes.^4^ Then, gene set enrichment analysis (GSEA) was performed and found that ubiquitin ligase-related signature gene sets were suppressed in the RR group (Fig. 1a and Supplementary Fig. 1c). Thus, to identify a critical factor for the acquisition of radioresistance, the expression levels of F-box E3 ligases were analyzed and found that FBXL14 strongly sensitizes NSCLC to radiation (Fig. 1b and Supplementary Fig. 1d–g). Moreover, FBXL14 was consistently lower in irradiate NSCLC than in nonirradiated NSCLC both in vitro and in vivo (Fig. 1c and Supplementary Fig. 1h–k). Kaplan–Meier survival analysis showed that lung cancer patients with high levels of *FBXL14* had longer survival times than those with low levels (Fig. 1d).

**Fig. 1:**
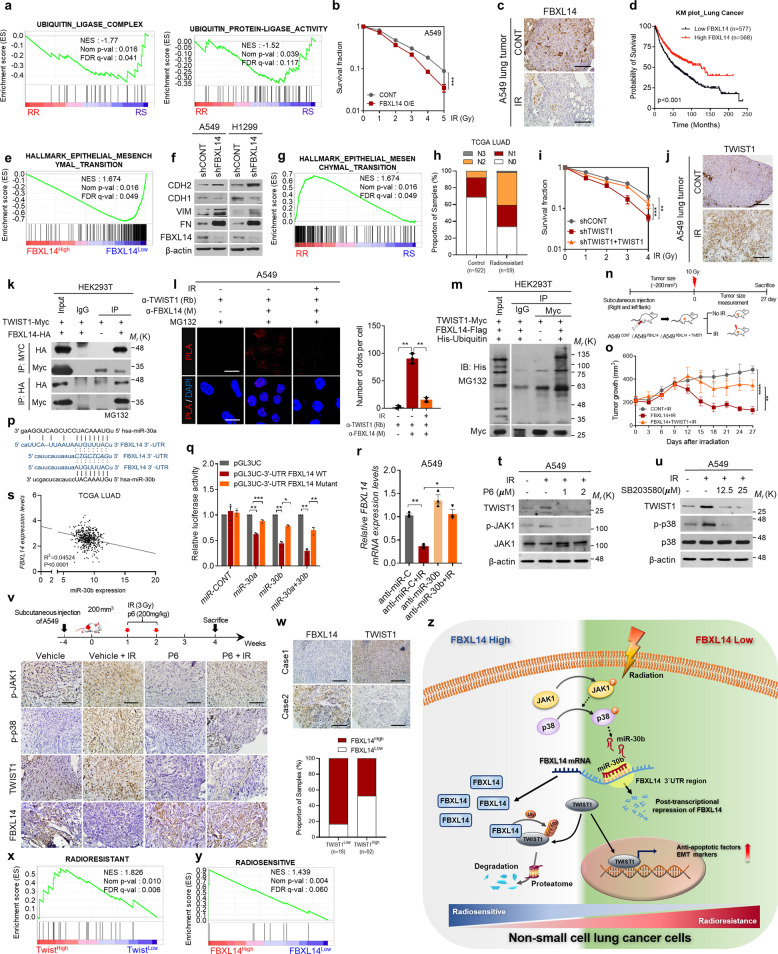
Loss of FBXL14 promotes mesenchymal shift and radioresistance of non-small cell lung cancer by TWIST1 stabilization. **a** GSEA of ubiquitin ligase complex and ubiquitin protein ligase activity gene signature in radioresistant versus radiosensitive NSCLC patients from GSE42127. NES normalized enrichment score, Nom *p* val normalized *p* value, FDR *q* val false discovery rate *q* value. **b** Clonogenic survival assays of A549 cells transfected with *FBXL14* or control empty vector prior to irradiation as indicated (*n* = 3 per group). **c** IHC analysis of FBXL14 in A549 xenograft tumors after radiation (*n* = 3 mice/group). Scale bar = 100 μm. **d** Kaplan–Meier survival analysis of lung cancer patients (http://kmplot.com/analysis); FBXL14 high versus low. **e** GSEA of hallmark epithelial to mesenchymal transition gene signature to *FBXL14* expression in NSCLC patients (GSE8894). **f** Western blot of EMT markers in NSCLC cells transduced with FBXL14 shRNA as indicated. **g** GSEA demonstrating enrichment of hallmark epithelial to mesenchymal transition gene signature in radioresistant versus radiosensitive NSCLC patients from GSE31210. **h** Lymph node stage analysis from TCGA NSCLC patients. **i** Clonogenic survival of A549 cells transduced with shTWIST1 prior to radiation (*n* = 3 per group). **j** IHC analysis in A549 xenograft tumors after treatment with IR (2 Gy/day × 5 days) or not (*n* = 3 mice/group). Scale bar: 100 μm. **k** Co-immunoprecipitation with Myc or HA antibody and western blot analysis to evaluate the interaction between TWIST1 and FBXL14 in HEK293T cells. **l** Representative images and quantification of in situ PLA showing the interaction between TWIST1 and FBXL14 both in A549 and H1299 cells. Scale bar: 100 μm. **m** Western blot analysis of ubiquitination of TWIST1 in HEK293T cells transfected with TWIST1-Myc, FBXL14-Flag, and/or His-ubiquitin as indicated. **n** Schematic illustration of animal experiment. **o** Effect of FBXL14 and/or TWIST1 in combination with IR on growth retardation of xenograft tumors formed by A549 cells. **p** Schematic diagram depicting recognition of hsa-miR-30a, -b for 3′-UTR of *FBXL14*, but not for the mutant form. **q** Luciferase reporter assay in HEK293T cells transfected with vectors encoding wild-type or mutant 3′-UTR of *FBXL14*, following the treatment with miRNA 0 as indicated. **r** RT-qPCR analysis of *FBXL14* expression levels in A549 cells pretreated with miR-30b inhibitor (anti-miR-30b) prior to radiation; levels were normalized to *ACTB*. **s** Pearson correlation analysis of *FBXL14* and miR-30b expression levels in TCGA lung adenocarcinoma patient cohort. **t**, **u** Western blot analysis of TWIST1 in A549 cells pretreated with P6 (**t**) or SB203580 (**u**) prior to radiation as indicated. **v** IHC analysis of p-JAK1, p-p38, TWIST1, and FBXL14 in xenograft tumor of mice, in which tumors were treated with radiation and/or P6 as indicated (*n* = 3 mice/group). Scale bar = 100 μm. **w** Representative images and contingency table for statistical analysis of IHC analysis showing the relationship between FBXL14 and TWIST1 in human lung cancer tissue array. Scale bar = 100 μm. **x** GSEA of “radioresistant” signature^4^ to *TWIST1* expression in NSCLC patients (GSE41271). **y** GSEA of “radiosensitive” signature to *FBXL14* expression in NSCLC patients (GSE8894). **z** Schematic illustration of the mechanism underlying stabilization of TWIST1 protein in response to radiation, leading to radioresistance in NSCLCs. Data are presented as mean ± SD and analyzed by Student’s *t* tests. *p < 0.05; **p < 0.01; ***p < 0.001

To determine the molecular function of FBXL14, GSEA was performed and found that EMT signature genes were negatively correlated with *FBXL14* expression (Fig. 1e). Consistent with the above analysis, knockdown of FBXL14 increased the EMT markers, and induced the acquisition of a more elongated and spindle-shaped morphology (Fig. 1f and Supplementary Fig. 2a, b). Emerging evidence suggests that EMT is induced in response to radiation,^5^ and we confirmed EMT markers were augmented after irradiation (Supplementary Fig. 2c–h). In contrast, FBXL14 overexpression suppressed radiation-induced EMT (Supplementary Fig. 2i–n).

Although EMT has been known as a cancer metastasis mechanism, several studies have suggested a correlation with radioresistance. However, how EMT program can associate the radioresistance is still unclear. Our analysis showed that EMT signature genes were highly expressed in the RR group of the various types of cancer patients (Fig. 1g and Supplementary Fig. 3). Furthermore, an increased proportion of patients with advanced lymph node stage accompanied recurrence after radiotherapy in TCGA lung cancer dataset (Fig. 1h). To investigate the critical regulator of radioresistance, EMT-TFs were silenced and analyzed clonogenic survival assay. Notably, TWIST1 depletion sensitized NSCLCs to radiation more strongly than the other EMT-TFs, and reconstitution of TWIST1 rescued the acquired radioresistance of these cells (Fig. 1i and Supplementary Fig. 4a–e). Furthermore, TWIST1 is critically increased in protein level after radiation (Fig. 1j and Supplementary Fig. 4f–o). Since FBXL14 was downregulated after irradiation, the correlation between TWIST1 and FBXL14 was investigated. Mechanistically, FBXL14 was directly interacted with TWIST1 and suppressed its protein stability (Fig. 1k–m and Supplementary Fig. 4p–t).

To unravel the mechanism underlying TWIST1-induced radioresistance, GSEA was performed and found that apoptosis signature genes were correlated with TWIST1 (Supplementary Fig. 5a). Among the antiapoptotic factors, *BIRC3*, *BCL2*, and *XIAP* were critically regulated by TWIST1 (Supplementary Fig. 5b–g). In addition, TWIST1 depletion enhanced the effect of radiation on the apoptosis of NSCLC (Supplementary Fig. 5h–q). Next, whether FBXL14 sensitizes NSCLC to radiation via TWIST1 downregulation was examined. The results of the clonogenic survival assay indicated that the increased radiosensitivity by FBXL14 overexpression was abolished by TWIST1 overexpression (Supplementary Fig. 6a, b). Consistent with our hypothesis, although radiation increased the expression of anti-apoptotic factors, FBXL14 overexpression attenuated the effect of radiation on these cells (Supplementary Fig. 6c–f). Moreover, knockdown of FBXL14 suppressed chemotherapy-induced apoptosis of NSCLC (Supplementary Fig. 6g). To validate our results in vivo, A549 cells transfected with FBXL14 and/or TWIST1 were subcutaneous injected into the flanks of male NSG mice (Fig. 1n). FBXL14 overexpression maximized the inhibitory effect of radiation on tumor growth; however, co-expression of TWIST1 recovered the tumor growth rate, which was decreased by FBXL14 combined with the radiation treatment (Fig. 1o and Supplementary Fig. 6h–m).

Next, the mechanism by which FBXL14 is downregulated following exposure to radiation was examined. Because no significant differences of DNA methylation in the FBXL14 promoter region following radiation were observed, the radiation inducible microRNAs (miRNAs) were investigated and choose candidates that can bind to 3′ untranslated region (UTR) of *FBXL14* mRNA, by using microRNA microarray (GSE101085) and target prediction algorithms (Supplementary Fig. 7a–c). From the predicted candidates, five microRNAs (miR-30a, miR-30b, miR-30c, miR-30d, and miR-30e) were selected (Supplementary Fig. 7d, e), and we investigated whether *FBXL14* mRNA levels are regulated by these miRNAs. Notably, both miR-30a and miR-30b significantly decreased *FBXL14* transcripts (Fig. 1p–r and Supplementary Fig. 7f–l). In addition, treatment with anti-miR-30b prevented the enhancing effect of radiation on TWIST1 protein stability, indicating that radiation increases TWIST1 accumulation through miR-30b-mediated FBXL14 depletion (Supplementary Fig. 7m–o) and miR-30b enhanced the acquisition of radioresistance in lung cancer (Supplementary Fig. 8). Importantly, *FBXL14* and miR-30b were negatively correlated in TCGA lung adenocarcinoma clinical cohort (Fig. 1s).

We next attempted to identify an upstream signaling regulator of miR-30b in response to radiation. Although radiation activated numerous intracellular signaling regulators, pretreatment with only SB203580 (p38 MAPK inhibitor) or P6 (pan-JAK inhibitor) attenuated the effect of radiation on the induction of miR-30b (Supplementary Fig. 9a, b). Coincident with the increase in miR-30b, FBXL14, TWIST1, and EMT markers were regulated by JAK and p38 (Fig. 1t, u and Supplementary Fig. 9c–h). Because JAK inhibition attenuated the activation of both JAK1 and p38 in A549 cells after radiation, we tested whether the inhibition of JAK1 attenuated the effect of radiation on p38 activation, FBXL14 downregulation, and the protein levels of TWIST1. Accordingly, A549 cells were injected subcutaneously into the BALB/c nude mice and revealed that the treatment with P6 attenuated radiation-induced p38 phosphorylation, FBXL14 downregulation, and miR-30b, TWIST1 upregulation (Fig. 1v and Supplementary Fig. 9i, k). In addition, JAK inhibition diminished the effect of radiation on EMT, but synergistically enhanced the effect of radiation on cancer cell apoptosis, as evidenced by the levels of CDH2 and cleaved caspase-3 (Supplementary Fig. 9l).

To evaluate the clinical importance of our findings, the negative correlation between TWIST1 and FBXL14 was validated in a human lung cancer tissue microarray (Fig. 1w). Although the expression level of *TWIST1* not exactly accords to its protein level, NSCLC patients (GSE31210 and GSE50081) with *TWIST1*^*High*^*FBXL14*^*Low*^ showed the worst survival rate (Supplementary Fig. 9m). Moreover, GSEA showed that NSCLC patients (GSE41271) with high *TWIST1* expression displayed transcriptomes similar to those of RR cells^4^ (Fig. 1x). In contrast, NSCLC patients (GSE8894) with high expression levels of *FBXL14* showed gene expression patterns had similar to those of RS cells^4^ (Fig. 1y).

In summary, our study identified FBXL14 as a novel factor for regulating the radiosensitivity of NSCLCs. Loss of FBXL14 following radiation leads to the accumulation of TWIST1, and subsequently promotes the acquisition of radioresistance through inducing EMT and antiapoptotic factors in NSCLCs (Fig. 1z). Altogether, our study highlights the regulatory mechanism of FBXL14 may be a therapeutic target to enhance the therapeutic efficacy of radiotherapy for lung cancer.

## Supplementary information

Supplementary Material clean
