# Supplementary material for: Loss of FBXL14 promotes mesenchymal shift and radioresistance of non-small cell lung cancer by TWIST1 stabilization
Source: Signal Transduct Target Ther. 2021 Jul 21;6:272. doi: 10.1038/s41392-021-00599-z (PMC8292372; doi:10.1038/s41392-021-00599-z)
Supplement: Supplementary file 1 — Supplementary Material clean [file 41392_2021_599_MOESM1_ESM.docx]

Supplementary Materials for

**Loss of FBXL14 promotes mesenchymal shift and radioresistance of non-small cell lung cancer by TWIST1 stabilization**

Yan-Hong Cui^1,2¶^, Jae-Hyeok Kang^1¶^, Yongjoon Suh^1^, Yi Zhao^1^, Joo Mi Yi^3^, In-Hwa Bae^4^, Hae-June Lee^5^, Dong Won Park^6^, Min-Jung Kim^7^*, Su-Jae Lee^1^*

Correspondence to: Su-Jae Lee (sj0420@hanyang.ac.kr) or Min-Jung Kim (kimmj74@kirams.re.kr)

**This PDF file includes:**

Extended Discussion

Materials and Methods

Supplementary Figures. S1 to S9

References

**Extended Discussion**

Radiotherapy is a standard treatment for patients with advanced-stage NSCLC.^1^ However, acquired radioresistance of NSCLC cells is the primary factor limiting treatment efficacy.^2^ Accordingly, numerous studies have examined the main determinants of radioresistance and its regulatory mechanisms to overcome therapy-induced resistance. However, the precise molecular mechanism driving radioresistance in NSCLCs remains unclear.

In this study, we show that FBXL14, an F-box E3 ligase family member, critically regulates radiosensitivity of NSCLC. An F-box subunit of the SCF complex, FBXL14 is the human homolog of the Partner of Paired (Ppa) gene product, and it is determined as tumor suppressor in breast cancer and glioma.^3, 4^ Here, we found that FBXL14 is downregulated in NSCLC cells after irradiation, leading to the acquired radioresistance of NSCLC cells by enhancing the accumulation of TWIST1. Emerging evidence suggests that radioresistance is strongly linked to epithelial-mesenchymal transition (EMT) in many solid cancers.^5, 6^ Via the EMT program, cancer cells lose their apical-basal polarity and intercellular junctions and acquire migration and invasion abilities. Accordingly, EMT has been accepted as a cellular mechanism underlying cancer metastasis.^7^ However, several strong studies have suggested that EMT is a complex cellular program driving multifaceted aspects of cancer progression, including the expansion of cancer stem cells, resistance to anticancer treatments, and metastasis.^8, 9^ Indeed, EMT inducers in cancer are entwined with a variety of oncogenic signaling pathways, endowing cancer cells with plasticity to adapt to pressures encountered during cancer progression.^6^ Despite its complexity, the EMT program is orchestrated by several EMT transcription factors (EMT-TFs), such as SNAIL, TWIST, and ZEB family members. Notably, their functions in driving EMT are often tissue-specific, and the proteins have additional roles.^10, 11^ Among the EMT TFs, we found that TWIST1 confers radioresistance on human NSCLC cells. After radiation, the TWIST1 protein accumulates in NSCLC cells, and induces the expression of the antiapoptotic factors BIRC3, XIAP and BCL2, primarily via its EMT-TF activity. Accordingly, our findings indicate that TWIST1 confers radioresistance on NSCLC cells in addition to its classical activity as an EMT inducer. Additionally, our data show that TWIST1 induces expression of antiapoptotic factors such as XIAP, BIRC3, and BCL2Previously, Zhang et al. reported an additional role for EMT-TF in which ZEB1 interacts with the USP7 deubiquitylase and enhances its ability to deubiquitylate and stabilize CHK1, thereby promoting DNA damage repair and radioresistance in breast cancer cells ^12^. Although EMT is widely accepted as a multifaceted cellular program underlying the resistance, stemness, and metastasis of cancer cells, our findings and those of previous studies suggest that these events are not caused by EMT itself; instead, they likely occur via EMT-TFs.

Importantly, our findings demonstrate that FBXL14 directly interacts with TWIST1 and catalyzes its ubiquitination for proteasomal degradation; however, radiation attenuates proteasomal degradation of TWIST1 via downregulation of FBXL14. A previous study showed that EMT-TFs, including TWIST1, are labile proteins and are regulated at the level of protein stability by the proteasomal system ^13, 14^. In a previous study, Ppa, F-box protein, was shown to degrade Snail in *Xenopus laevis* in a phosphorylation-independent manner ^15^. Similarly, FBXL14 promotes the degradation of EMT-TFs, including TWIST1, in mammalian cells^16^, supporting our observations. Thus, the regulatory mechanisms of F-box E3 ligases have attracted research attention. In this study, we showed that radiation-induced activation of JAK1/p38 promotes an increase in the level of miR30b, which directly targets the *FBXL14* transcript in NSCLC cells. In confirmation of this signaling axis, targeting either JAK1 or miR30b attenuated the effect of radiation on TWIST1-associated radioresistance as well as EMT. Considering the importance of radioresistance in clinical outcomes, our findings reveal the underlying mechanism by which the TWIST1 protein is upregulated posttranslationally in response to radiation.

In summary, high expression of TWIST1 has been widely detected in human lung cancers ^17, 18^ and other cancer types ^19, 20^. Our findings indicate that loss of FBXL14 by radiation treatment leads accumulation of TWIST1 and subsequently induces radioresistance. As depletion of TWIST1 can endow NSCLC cells with radiosensitivity *in vitro* and *in vivo*, we suggest that TWIST1-targeting agents can be used as radiosensitizers in NSCLC. In conclusion, this study reveals the regulatory mechanism underlying the acquisition of adaptive radioresistance and induction of EMT by TWIST1 stabilization, which is mediated by the JAK1/p38 MAPK/miR30b/FBXL14 signaling axis after irradiation.

**Materials and Methods**

**Chemical reagents and antibodies**

Caspase-3/7 reagent was obtained from Promega (Madison, WI, USA). Collagen Type I was purchased from Corning Costar (Corning, NY, USA). CHX, MG132, U0126, SP600125, SB203580, WP1066, and P6 were obtained from Calbiochem (San Diego, CA, USA). Monoclonal antibodies specific for HA and Myc and polyclonal antibodies specific for VIM (sc-6260), STAT3 (sc-482), SNAI2 (sc-10437), JAK1 (sc-277), p-JAK1 (sc-16773), FBXO15 (sc-84823), TWIST1 (sc-15393), and ubiquitin (sc-58450) were purchased from Santa Cruz Biotechnology (Dallas, TX, USA). Antibodies specific for CDH2 (#610920), PARP (#611038), and annexin-V-FITC (#556419) were purchased from BD Biosciences (San Jose, CA, USA). Antibodies specific for p-STAT3 (Y705) (#9131), JNK (#9252), cleaved caspase-3 (#9661), SNAI1 (#3879), p-ERK (#4377), p-JNK (#4668), p-P38 (#4511), P38 (#8690), JNK (#9252), and ERK (#9102) were purchased from Cell Signaling Technology (Danvers, MA, USA). The monoclonal antibody specific for the His tag (ab16184) was obtained from Abcam (Cambridge, UK). Polyclonal antibodies specific for FBXL14 (#HPA053889) and β-actin (#A5441) and the monoclonal antibody specific for ZEB1 (#HPA027524) were obtained from Sigma (St. Louis, MO, USA).

**Cell culture**

A549 and H1299 human lung adenocarcinoma (LUAD) cells were purchased from the American Type Culture Collection (Manassas, VA, USA). H460 human LUAD cells, HCC95 and SNU1330 human lung squamous cell carcinoma (LSCC) cells, and HEK293T cells were purchased from the Korean Cell Line Bank. HEK293T cells were grown in DMEM, while A549, H1299, H460, HCC95, and SNU1330 cells were grown in RPMI medium. All media were supplemented with 10% fetal bovine serum, penicillin (100 U/mL), and streptomycin (100 μg/mL). Tissue culture reagents were purchased from Gibco (Grand Island, NY, USA). All cell lines were negative for mycoplasma contamination and were not passaged for longer than 6 months after thawing. All cell culture media, fetal bovine serum, penicillin/streptomycin, and trypsin were purchased from Gibco.

**Radiation**

NSCLC cells were exposed to radiation using a ^137^Cs γ-ray source (Atomic Energy of Canada, Ltd., Mississauga, ON, Canada) at a dose rate of 3.81 Gy/min. Mice bearing lung tumors were locally irradiated with the same γ-ray source.

**RT-qPCR**

Total RNA was isolated manually using TRIzol (Invitrogen, Carlsbad, CA, USA). All RT-qPCRs were performed using a KAPA SYBR FAST qPCR kit from KAPA Biosystems (Wilmington, MA, USA) according to the manufacturer’s instructions. Reactions were carried out in a Rotor Gene Q thermocycler (Qiagen, Hilden, Germany). Results are expressed as fold change values relative to the control sample as calculated by the ΔΔCt method. *GAPDH* was used as the internal normalization control.

**Western blot analysis**

Cell lysates were prepared by extracting proteins with lysis buffer [40 mM Tris–HCl (pH 8.0), 120 mM NaCl, 0.1% Nonidet-P40] supplemented with protease inhibitors. Proteins were separated by SDS-PAGE and transferred to a nitrocellulose membrane (Amersham plc, Amersham, UK). The membrane was blocked with 5% nonfat dry milk in Tris-buffered saline and incubated with primary antibodies overnight at 4°C. Membranes were reacted with a peroxidase-conjugated secondary antibody, and proteins were visualized by enhanced chemiluminescence procedures (Amersham) according to the manufacturer’s protocol.

**Methylation-specific PCR**

Genomic DNA was isolated using standard phenol/chloroform methods and subjected to bisulphite modification with an EZ DNA Methylation Kit (Zymo Research, Irvine, CA, USA). Methylation-specific PCR was performed for the *FBXL14* promoter region using 1 μL of modified DNA as a template and JumpStart Red Taq DNA Polymerase (Sigma) as previously described.^21^ Bisulphite sequencing primers were designed using Meth Primer (http://www.urogene.org/cgi-bin/methprimer/methprimer.cgi) and had the following sequences: forward, 5′-TTTTAATAGTAAAGAAAAAGAAGGAA-3′; reverse, 5′-ACCCCTAATTTTTAACCCTATAAAC-3′. The amplicons were gel-purified and subcloned into the pCRII-TOPO vector (Invitrogen). Clones were randomly selected and sequenced on an ABI3730xl DNA analyser to determine the methylation patterns of each locus.

**Plasmid constructs and transfection**

The lentiviral pLKO.1-shCont, pLKO.1-shFBLX14, and pLKO.1-shTWIST1 vectors were purchased from Sigma-Aldrich. The pCMV6-TWIST1-Myc-FLAG and pCMV6-FBXO15-Myc-FLAG vectors were purchased from Origene Technologies, Inc. (Rockville, MD, USA). For preparation of the reporter construct, a fragment of the human FBXL14 3′ UTR containing the miR-30a/miR-30b binding site (ATGTTTAC) was purchased from Integrated DNA Technologies (Coralville, IA, USA). The human FBXL14 3′ UTR mutant (CAGCUCAG) fragment was generated by site-directed mutagenesis. Both the resulting DNA fragments and the pGL3uc vector were digested with XbaI and PstI and ligated to generate the pGL3uc-FBXL14 3′ UTR plasmid.

Cells were transfected with DNA vectors using Lipofectamine and Plus reagents (Invitrogen) according to the manufacturer’s instructions. All siRNAs, miRNA inhibitors, and mimics were purchased from Genolution Pharmaceuticals, Inc. (Seoul, Korea) and introduced into cells using Lipofectamine 2000. The transfection efficacy was confirmed by western blotting and/or RT-qPCR.

**Transduction**

For lentivirus production, HEK293T cells were transduced with the lentiviral packaging plasmids pLP1, pLP2, and pLP/VSV-G along with a shRNA lentiviral vector. Forty-eight hours after transfection, the viral supernatant was collected and passed through a 0.45-μm filter. The viral supernatant was then supplemented with 8 μg/mL polybrene (Sigma) and used for transduction. Transduced cells were selected after 2 weeks of growth in medium containing puromycin (2 µg/mL).

**Luciferase reporter assay**

Luciferase reporter assays were performed using vectors encoding putative 3′ UTR target sites. In brief, HEK293T cells were seeded into 60-mm dishes. At ~50% confluence, cells were cotransfected with reporter plasmid (1 µg), pRL-CMV-Renilla (Promega) plasmid (1 µg), and miRNA using Lipofectamine and Plus reagents (Invitrogen) for 48 h. Firefly luciferase activity was measured using a dual-luciferase reporter assay system (Promega) according to the manufacturer’s instructions and was normalized to Renilla luciferase activity. All experiments were performed in triplicate.

**Coimmunoprecipitation**

HEK293T cells were cotransfected with TWIST1, HA-tagged FBXL14 using Lipofectamine transfection reagent. Two days after transfection, cells were treated with MG132 (10 μM) for 6 h, harvested, and lysed in cold lysis buffer supplemented with protease inhibitors. Cell lysates were precleared with protein A-Sepharose (Sigma-Aldrich), and the resulting supernatant fractions were incubated with primary antibodies at 4°C for 12 h. Immunoprecipitates were subsequently collected by incubation with protein A-Sepharose for 2 h followed by centrifugation for 2 min at 4°C. Pellets were then washed three times with lysis buffer. Immunoprecipitates dissolved in SDS sample buffer were analyzed by western blotting.

**CHX pulse-chase assays**

Pulse-chase assays were performed as previously described.^22^ Two days after transfection, cells were treated with 100 μg/mL CHX dissolved in dimethyl sulfoxide, harvested at different times, and subjected to TWIST1 immunoblotting.

**Clonogenic survival assay**

Cells (10^3^ cells/60-mm dish) were irradiated with a ^137^Cs γ-ray source and cultured for 10–14 days. The resulting colonies were fixed with methanol:acetic acid (10:1, v/v) and stained with 0.8% Coomassie blue.

**Cell death assay**

Apoptotic cell death was detected by fluorescence-activated cell sorting (FACS) to assess propidium iodide staining, annexin-V staining, and caspase-3/7 activity. A total of 10,000 events per sample were analyzed in a BD FACS Calibur using Cell Quest software (BD Biosciences).

**Immunocytochemistry**

Cells were fixed with 4% paraformaldehyde, permeabilized, and incubated with the appropriate primary antibodies in phosphate-buffered saline (PBS) containing 1% bovine serum albumin and 0.1% Triton X-100 at 4°C overnight. The following primary antibodies and dilutions were used: anti-human TWIST1 (1:200), anti-CDH2 (1:200), anti-Myc (1:200), anti-VIM (1:200), anti-HA (1:200), and anti-FBXL14 (1:200). Staining was visualized using anti-rabbit or anti-mouse Alexa Fluor 488-conjugated and anti-rabbit or anti-mouse Alexa Fluor 546-conjugated antibodies (Molecular Probes, Eugene, OR, USA). Nuclei were counterstained with DAPI (Sigma), and cells were imaged at 60× magnification with a Nikon confocal fluorescence microscope (Tokyo, Japan).

***In situ* PLA**

Cells cultured on a cover slip were treated with 10 μM MG132 for 6 h and fixed with 4% paraformaldehyde. Fixed cells were permeabilized with 0.1% Triton X-100 and 10% fetal bovine serum in PBS before incubation with anti-TWIST1 (1:200) and anti-FBXL14 (1:200) antibodies at 4°C overnight. *In situ* PLA was performed according to the manufacturer’s protocol using a Duolink Detection Kit (Sigma) with a pair of nucleotide-labeled secondary antibodies. Following ligation and amplification of the PLA probes, signals were analyzed by confocal microscopy and quantified using ImageJ software (NIH, Bethesda, MD, USA).

**IHC analysis**

Mouse and patient tissues were fixed with formalin and embedded in paraffin. Tissue sections were subsequently deparaffinized in xylene, rehydrated through a standard ethanol gradient, and treated with PBS. Epitopes were unmasked with 20 mg/mL proteinase K in PBS containing 0.1% Triton X-100. Sections were stained with hematoxylin & eosin (H&E) or immunostained overnight at 4°C. After washing with PBS, sections were incubated with biotinylated goat anti-rabbit IgG or anti-mouse IgG for 30 min. After another wash with PBS, sections were treated with ABC reagent (Vector Laboratories, Inc, Burlingame, CA, USA) for 30 min, counterstained with hematoxylin, cleared through a graded ethanol series and xylene, and finally mounted with Canada balsam. Images were acquired with a DP71 digital imaging system connected to an IX71 microscope (Olympus, Tokyo, Japan). IHC scores were calculated as the mean intensity values from three randomly selected fields.

**Migration and invasion assays**

For the invasion assay, cells (1 × 10^5^) were seeded in Transwell chambers containing 8-μm pore size filter inserts (Corning, Inc.) with the upper surface of the filter membrane precoated with 10 mg/mL growth factor-reduced Matrigel (BD Biosciences). The lower chambers were filled with 0.8 mL of growth medium. After incubation for 24 h at 37°C, noninvaded cells on the upper surface of the filter membrane were removed with a cotton swab, and invaded cells on the lower surface were fixed and stained with a Diff-Quick kit (Thermo Fisher Scientific, Waltham, MA, USA). For the migration assay, we used Transwells with inserts containing the same type of membrane but without the Matrigel coating. Migration and invasion were quantified by counting the cells in five microscopic fields per well, and data are expressed as the average number of cells per microscopic field.

**Animal experiments**

A549 cells (1 × 10^6^) were injected subcutaneously into the flanks of 10-week-old male NSG mice (n = 8 mice/group in Supplementary Fig. 5l-q; n = 5 mice/group in Fig. 1n-o and Supplementary Fig. 6h-m; Jackson Laboratories, Bar Harbor, ME, USA). At a volume of 200 mm^3^ (Supplementary Fig. 5l-q) or 300 mm^3^ (Fig. 1n-o and Supplementary Fig. 6h-m), the xenograft tumors were locally irradiated with 10 Gy or left untreated as controls. Tumor growth was then monitored every 3 days. Mice were sacrificed, and the tumors were measured, and weighed, and subsequently analyzed by IHC and RT-qPCR. In addition, A549 cells (1 × 10^6^) were injected into the tail veins of 8-week-old female athymic BALB/c nude mice (n = 8; Orient, Gyeonggido, Korea) and allowed to form tumors in the lungs. When lung tumors had formed, the whole lungs were exposed locally to fractionated radiation (2 Gy/day×5 days) or nonirradiated as a control (n = 3 mice/group in Fig. 1c and Supplementary Fig. 1g, 2m, n). Mice were sacrificed, and lung tissues were sectioned for IHC analysis. In a further experiment, A549 cells (1 × 10^6^) were subcutaneously injected into the right flank of 8-week-old female athymic BALB/c nude mice (n = 3 mice/group in Fig. 1v and Supplementary Fig. 9i-l). At a volume of 200 mm^3^, the tumors were treated with local radiation (3 Gy/week×2 weeks) and/or P6 (a pan-JAK inhibitor; 200 mg/kg). Mice were then sacrificed two weeks after treatment, and the xenograft tumors were sectioned for IHC analysis. Tumor formation and size were monitored by sacrificing one or two mice per group. All animal housing and experimental procedures were approved by the Institutional Animal Care and Use Committee of the Center for Laboratory Animal Sciences, Medical Research Coordinating Center, and by the HYU Industry-University Cooperation Foundation.

**Gene microarray analysis**

To investigate radiation-induced miRNA expression changes in A549 cells, miRNA microarray analysis was conducted with a SurePrint G3 Human v16 miRNA 8x60K microarray (Agilent Technologies, Santa Clara, CA, USA) as previously described.^23^ In brief, total RNA was extracted from each group of cells using TRIzol. RNA samples with integrity values of >8.0 and purity values (A260/280 and A260/A230) of >1.8 were subjected to miRNA microarray analysis. The intensities were measured using an Agilent SureScan microarray scanner (Agilent Technologies), and numerical data were extracted using Agilent Feature Extraction software (version 10.7). The extracted data were then filtered using flag-present and *t*-tests, and miRNAs showing a >2.0-fold increase or decrease in expression were selected for further analysis. For GSEA, normalized expression data were analyzed and visualized with GSEA software (version 4.0.3, http://www.broadinstitute.org/gsea).

**Human tissue microarray and Kaplan-Meier analysis**

NSCLC tissue microarray samples were obtained from US Biomax (LC121a; Derwood, MD, USA) and reviewed by a pathologist to confirm the lung carcinoma diagnosis, histological grade, and tumor purity. Survival data were obtained from an online Kaplan-Meier database (http://kmplot.com/analysis).^24^

**Statistical analysis**

All data are presented as the means ± standard deviations (SDs). Each experiment was repeated at least three times. Statistical analyses were conducted with two-tailed parametric Student’s *t*-tests or analysis of variance for multivariate analysis in GraphPad Prism software 7.0 (GraphPad, Inc., La Jolla, CA, USA). The variance was similar between groups, and *p* values of <0.05 were considered significant. Significance was defined as **P* < 0.05, ***P* < 0.01, and ****P* < 0.001 compared to controls. Nonsignificance was denoted as n.s.

**DATA AND SOFTWARE AVAILABILITY**

The miRNA microarray dataset produced in this study is deposited in the NCBI Gene Expression Omnibus (http://ncbi.nlm.nih.gov/geo) and accessible under GEO series accession number GSE101085 (**Supplementary Fig. 7b**).

Other datasets used in this study are publicly available in UCSC Xena (http://xena.ucsc.edu; TCGA lung cancer) and GEO database (GSE31210, GSE41271, GSE50081, GSE116959, GSE87211, GSE9712, GSE7696, and GSE8894).


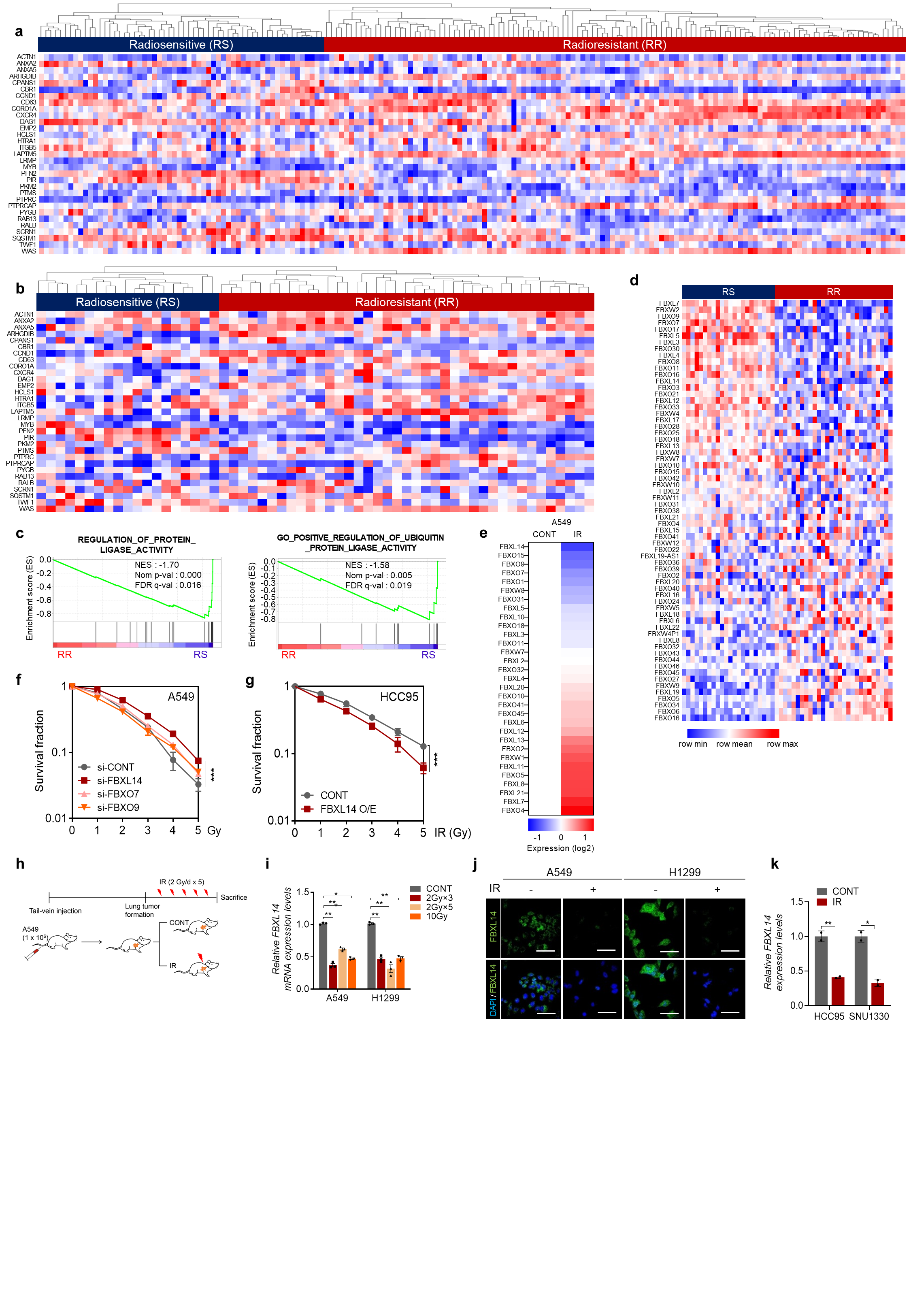


**Supplementary Fig. 1 Downregulation of E3 ligase FBXL14 enhances radioresistance of NSCLC cells.** **a-b** Hierarchical clustering was used to determine the expression pattern of 31-gene signature on the sample from GSE42127 **(a)** and GSE10072 **(b)**. **c** GSEA of ubiquitin ligase complex and ubiquitin protein ligase activity gene signature in radioresistant versus radiosensitive NSCLC patients from GSE10072. NES, normalized enrichment score; Nom p-val, normalized p-value; FDR q-val, false discovery rate q-value. **d** Heatmap of NSCLC patients (GSE27262) divided by hierarchical clustering, showing expression levels of F-box E3ligases. **e** RT-qPCR analysis of expression levels of F-box E3 ligases in A549 cells after radiation. **f** Clonogenic survival assays of A549 cells transduced with siRNAs targeting FBXL14, FBXO7, or FBXO9 prior to IR as indicated (n = 3 per group). **g** Clonogenic survival assays of HCC95 cells transfected with FBXL14 or control empty vector prior to irradiation as indicated (n = 3 per group). **h** Schematic illustration of animal experiment (n = 3 mice/group). **i** RT-qPCR analysis of *FBXL14* expression in LUAD cells after radiation as indicated. **j** ICC of FBXL14 in LUAD cells after radiation (2Gy/d×3). Scale bar = 100 μm. **k** RT-qPCR analysis of *FBXL14* expression in LSCC cells after radiation (2Gy/d×3).

Data are presented as mean ± SD and analyzed by Student’s t-tests. **p* < 0.05; ***p* < 0.01; ****p* < 0.001


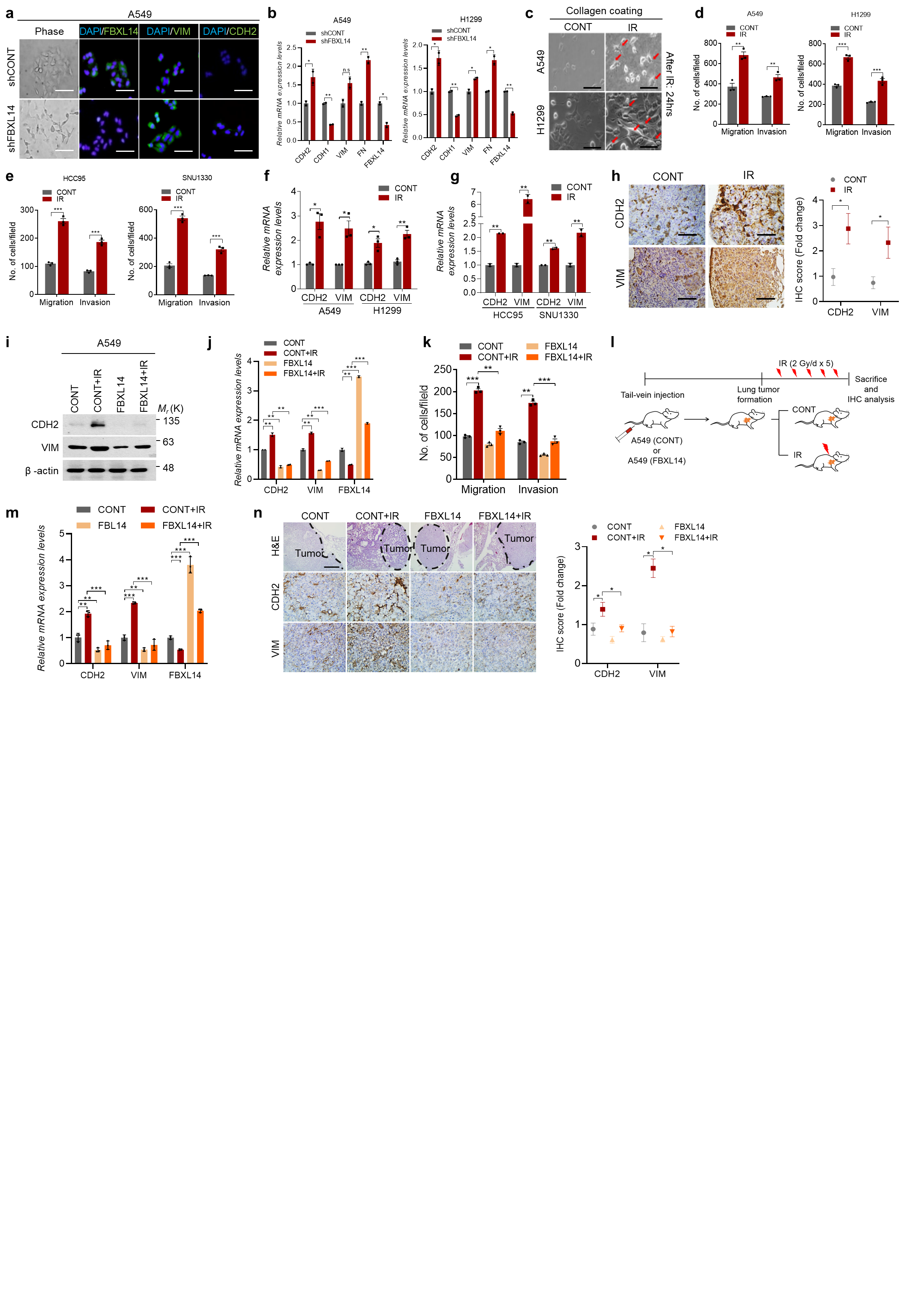


**Supplementary Fig. 2 FBXL14 suppresses EMT of NSCLCs.** **a** ICC analysis of EMT markers in A549 cells transduced with FBXL14 shRNA as indicated. Scale bar = 100 μm. **b** RT-qPCR of EMT markers and FBXL14 in LUAD cells transduced with FBXL14 shRNA. **c** Collagen–based morphological changes were observed both in A549 and H1299 cells after irradiation. Scale bar = 100 μm. **d** Transwell migration and invasion assays of LUAD cells after irradiation. **e** Transwell migration and invasion assays of LSCC cells after irradiation. **f** RT-qPCR analysis of *CDH2* and *VIM* in LUAD cells after radiation. **g** RT-qPCR analysis of *CDH2* and *VIM* in LSCC cells after radiation. **h** Representative IHC images of CDH2 and VIM in irradiated/non-irradiated xenograft tumors formed by A549 cells. Scale bar = 100 μm. **i** Western blot analysis of CDH2 and VIM in A549 cells transfected with FBXL14 prior to radiation as indicated. **j** RT-qPCR of CDH2, VIM, and FBXL14 in A549 cells transfected with FBXL14 prior to radiation as indicated. **k** Transwell migration and invasion assays of A549 cells transfected with FBXL14 prior to IR. **l** Schematic illustration of animal experiment (n=3 mice/group). **m** RT-qPCR of EMT markers and FBXL14 in irradiated/non irradiated lung tumors formed by A549cells after tail-vein injection into BALB/c nude mice. **n** IHC analysis of EMT markers in irradiated/non-irradiated lung tumors. Scale bar = 100 μm.

Data are presented as mean ± SD and analyzed by Student’s t-tests. **p* < 0.05; ***p* < 0.01; ****p* < 0.001


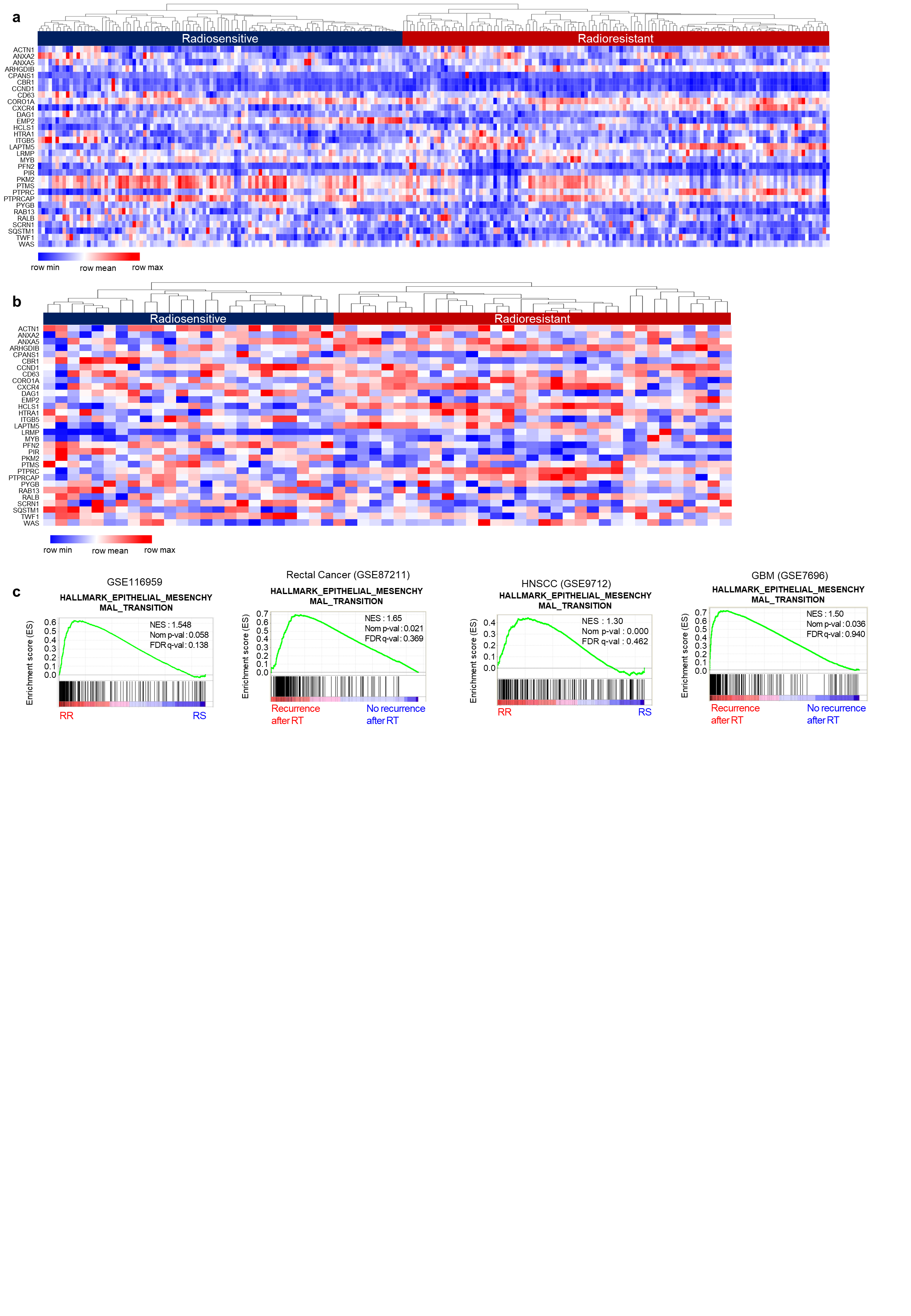


**Supplementary Fig. 3 Radioresistant cancer patients express high level of EMT signature genes.** **a-b** Hierarchical clustering was used to determine the expression pattern of 31-gene signature on the sample from GSE31210 **(a)** and GSE116959 **(b)**. **c** GSEA demonstrating enrichment of hallmark epithelial to mesenchymal transition gene signature in radioresistant versus radiosensitive rectal cancer (GSE87211), HNSCC (GSE9712) and GBM (GSE7696) patients.


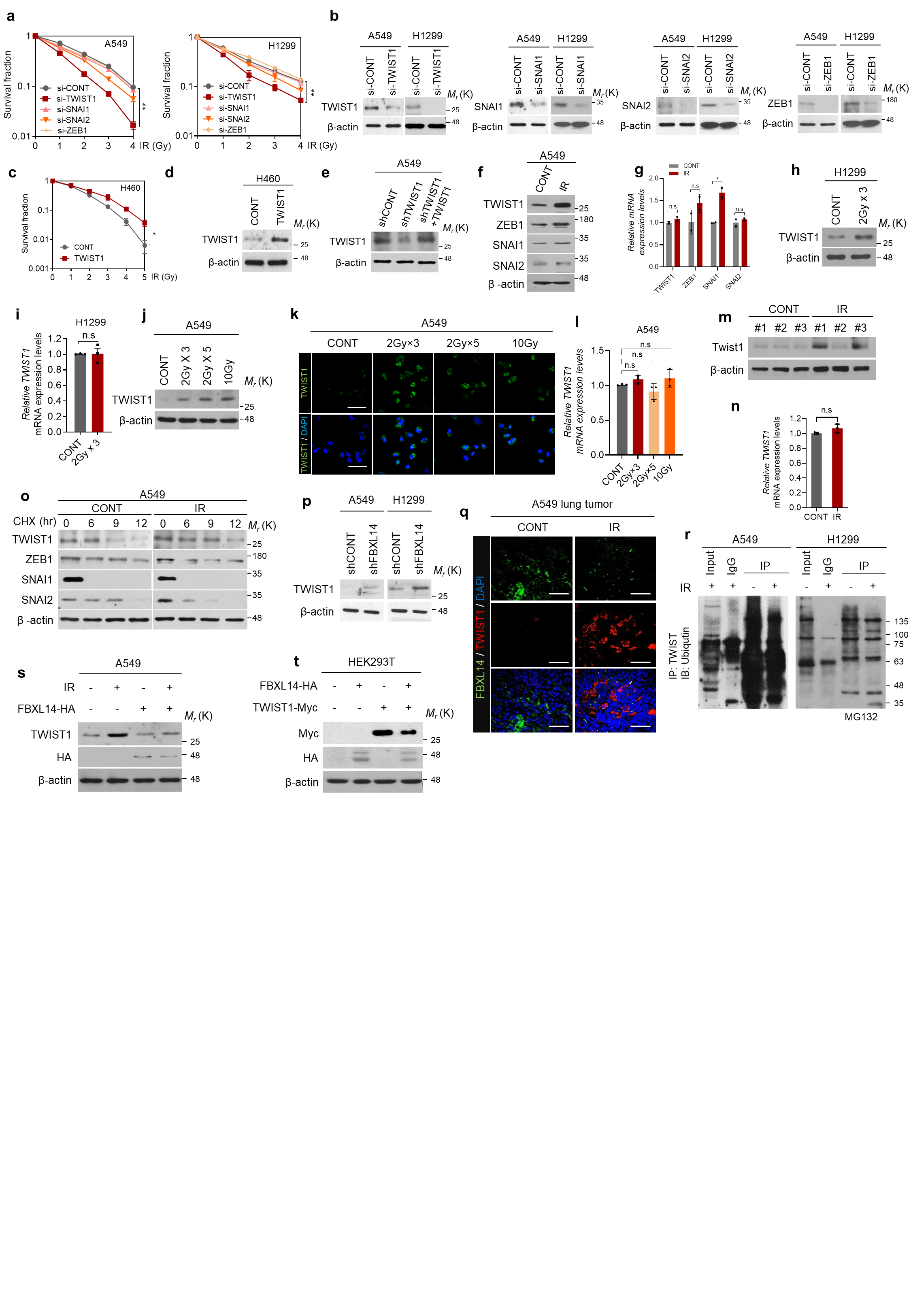


**Supplementary Fig. 4 Radiation enhances TWIST1 protein stability by downregulating FBXL14 E3 ligase expression.** **a** Clonogenic survival assays of A549 and H1299 cells transduced with siRNAs targeting TWIST1, SNAI1, SNAI2, or ZEB1 prior to IR as indicated (n = 3 per group). **b** Immunoblotting of TWIST1, SNAI1, SNAI2 and ZEB1 in both A549 and H1299 cells transfected with siRNA as indicated. β-actin was used as a normalization control. **c** Clonogenic survival of H460 cells transfected with *TWIST1* or control empty vector prior to irradiation as indicated (n = 3 per group). **d** Western blot analysis for validation of TWIST1 overexpression in H460 cells after transfection. **e** Western blot analysis of TWIST1 in A549 cells as indicated. **f-g** Western blot **(f)** and RT-qPCR **(g)** of EMT-TFs in A549 cells after radiation (10 Gy). **h-i** Western blot (**h**) and RT-qPCR (**i**) analysis of TWIST1 in H1299 cells after radiation as indicated. **j-k** Western blot (**j**) and ICC analysis (**k**) of TWIST1 in A549 cells after radiation as indicated. Scale bar = 100 μm. **l** RT-qPCR of *TWIST1* expression in A549 cells after 2 Gy/day×3 days, 2 Gy/day×5 days or single dose 10 Gy. **m-n** Western blot **(m)** and RT-qPCR **(n)** of TWIST1 in A549 xenograft tumors after treatment with IR (2 Gy/day×5 days) or not. **o** Western blot analysis of the half-life of EMT-TFs in A549 cells irradiated or non-irradiated followed by treatment with CHX. **p** Western blot analysis of TWIST1 in NSCLC cells transduced with shCONT or shFBXL14. **q** IHC analysis of TWIST1 and FBXL14 in A549 xenograft tumors. Scale bar: 100 μm. **r** Immunoprecipitation with TWIST1 antibody and western blot analysis of ubiquitination of TWIST1 in A549 and H1299 cells after radiation. **s** Western blot analysis of TWIST1 in A549 cells after transfection with FBXL14-HA prior to IR. **t** Western blot analysis of Myc and HA in HEK293T cells transfected with either FBXL14-HA or TWIST1-Myc, or co-transfected with both.

Data are presented as mean ± SD and analyzed by Student’s t-tests. **p* < 0.05; ***p* < 0.01; ****p* < 0.001


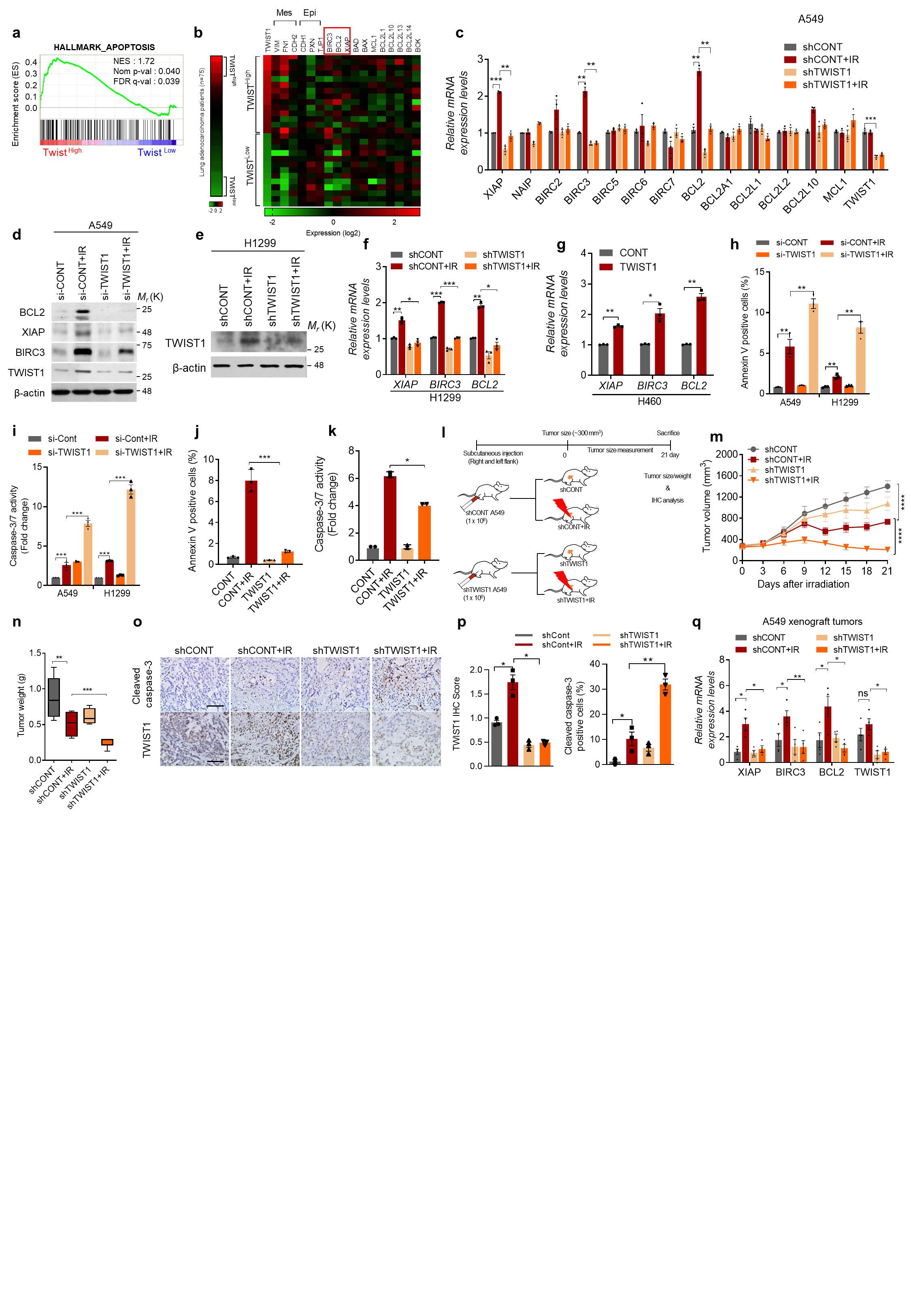


**Supplementary Fig. 5 TWIST1 enhances the radioresistance of NSCLCs by upregulating anti-apoptotic factors.** **a** GSEA demonstrating enrichment of hallmark apoptosis gene signature in TWIST1^High^ versus TWIST1^Low^ NSCLC patients from GSE41271. **b** Heatmap of lung adenocarcinoma patients (GSE12667) ranked with TWIST1 expression, showing expression levels of EMT markers (Mes, mesenchymal; Epi, epithelial) and anti-apoptotic genes between TWIST1^High^ and TWIST1^Low^ lung adenocarcinoma patients. **c** RT-qPCR analysis of antiapoptotic gene expression in A549 cells transduced with TWIST1 shRNA (shTWIST1) or negative control shRNA (shCONT) prior to radiation as indicated. **d** Western blot analysis of antiapoptotic protein levels in A549 cells transfected with TWIST1 siRNA prior to IR (10 Gy). **e** Western blot of TWIST1 in H1299 cells transduced with shRNA prior to radiation. **f** RT-qPCR analysis of anti-apoptotic gene expression in H1299 cells transduced with shTWIST1 prior to radiation. **g** RT-qPCR analysis of XIAP, BIRC3, and BCL2 expression in TWIST1-expressing H460 cells. **h-i** Quantification of apoptotic cell death by FACS analysis using annexin-V staining (**h**) and caspase-3/7 activity (**i**) in A549 and H1299 cells transfected with each siRNA following IR (10 Gy). **j-k** Quantification of apoptotic cell death by FACS analysis using annexin-V staining **(j)** and caspase-3/7 activity **(k)** in H460 cells transfected with TWIST1 following IR (10 Gy). **l** Schematic illustration of animal experiment (n = 8 mice/group). **m-n** Tumor growth **(m)** and weight **(n)** formed by subcutaneous injection of A549 cells transduced with shRNA targeting TWIST1 (shTWIST1) or control shRNA (shCONT). **o** IHC analysis of cleaved caspase-3 and TWIST1 in xenograft tumors formed by A549 cells as indicated. Scale bar = 100 μm. **p** Graphical depiction of IHC analysis for TWIST1 and cleaved caspase-3. **q** RT-qPCR analysis of *XIAP*, *BIRC3*, and *BCL2* expression in xenograft tumors by A549 cells as indicated.

Data are presented as mean ± SD and analyzed by Student’s t-tests. **p* < 0.05; ***p* < 0.01; ****p* < 0.001


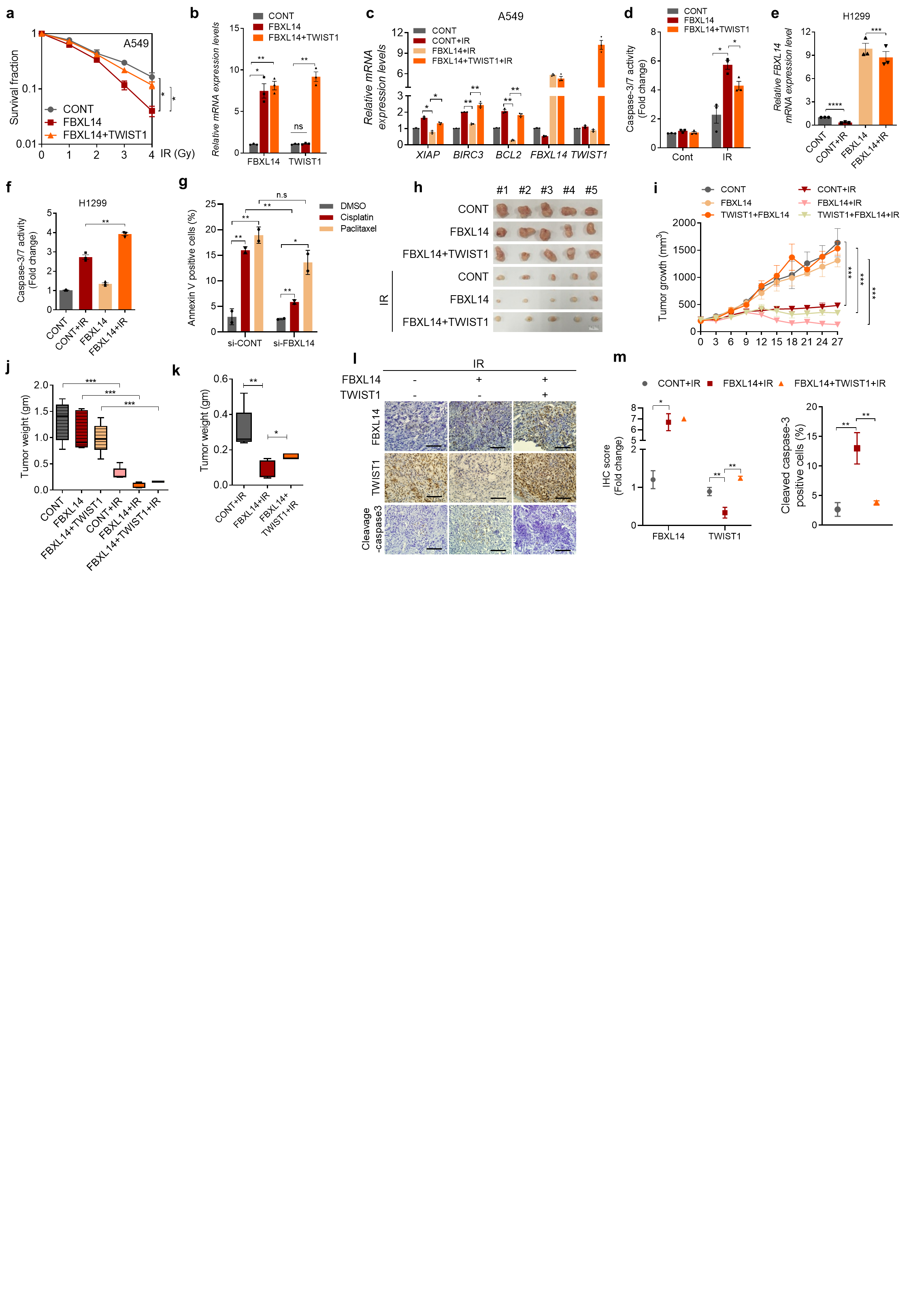


**Supplementary Fig. 6 FBXL14 sensitizes NSCLCs to radiation by downregulation of TWIST1.** **a** Clonogenic survival assays of A549 cells transfected with FBXL14 alone or in combination with TWIST1 prior to IR as indicated (n = 3 per group). **b** RT-qPCR analysis of *FBXL14* and *TWIST1* in A549 cells after transfection as indicated. **c** RT-qPCR analysis of XIAP, BIRC3, BCL2, FBXL14, and TWIST1 expression in A549 cells transfected with FBXL14 and/or TWIST1 prior to IR (10Gy). **d** FACS analysis of caspase-3/7 activity in A549 cells transfected with FBXL14 alone or in combination with TWIST1 prior to IR. **e** RT-qPCR analysis of *FBXL14* in H1299 cells transfected with either empty vector (CONT) or FBXL14 prior radiation. **f** FACS analysis for caspase-3/7 activity in H1299 cells transfected with CONT or FBXL14 prior to radiation. **g** FACS analysis using annexin-V staining in A549 cells transfected with FBXL14 siRNA prior to treatment with cisplatin (50μM) or paclitaxel (5μM). **h** Tumor images after sacrifice of mice in **Fig. 1n** (n = 5 mice/group). **i** Effect of radiation on growth retardation of xenograft tumors formed by A549 cells expressing FBXL14 alone or in combination with TWIST1. **j** Tumor weight after sacrifice of mice in **(h)**. **k** Tumor weights upon sacrifice of mice in **Fig. 1o**. **l** IHC analysis in xenograft tumor tissues as indicated. Scale bar = 100 μm. **m** Graphical depiction of IHC analysis in **(l)**.

Data are presented as mean ± SD and analyzed by Student’s t-tests. **p* < 0.05; ***p* < 0.01; ****p* < 0.001


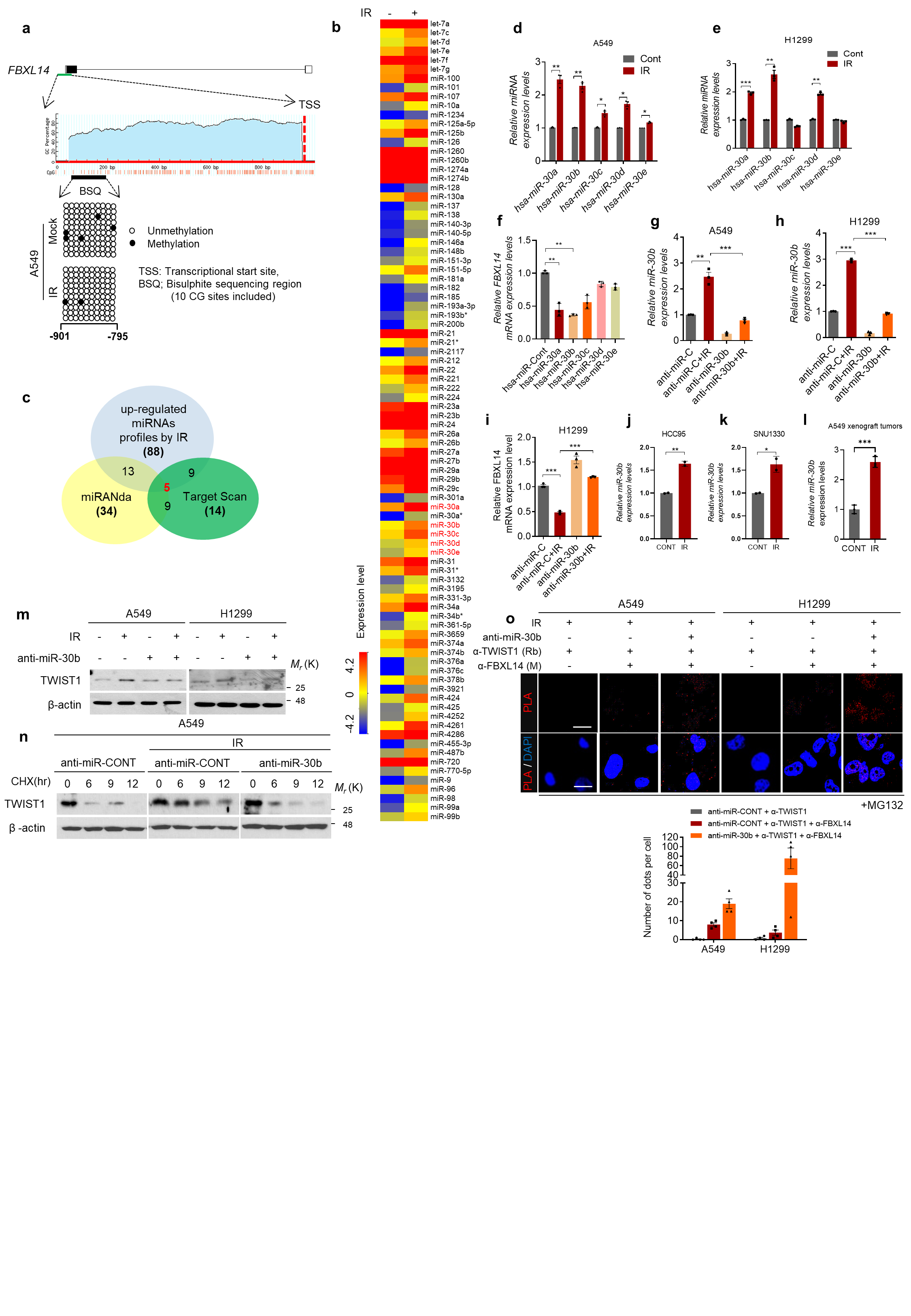


**Supplementary Fig. 7 The FBXL14 E3 ligase is regulated by radiation-inducible miR-30b.** **a** DNA methylation analysis in the CpG island of the *FBXL14* promoter using bisulfite sequencing in genomic DNA isolated from A549 cells after radiation (2 Gy/day×3 days). **b** Heatmap of miRNA microarray analysis of A549 cells irradiated (2 Gy/day×3 days) or non-irradiated. **c** Venn diagram showing the number of miRNA upregulated after IR, and identified as potential miRNA against *FBXL14* in two different mRNA target-predicting algorithms; miRANda and Target Scan. **d-e** RT-qPCR analysis of miR-30 family members in A549 **(d)** and H1299 **(e)** cells after IR (2 Gy/day×3 days). **f** RT-qPCR analysis of *FBXL14* in A549 cells after treatment with miR-30 family member mimics as indicated. **g-h** RT-qPCR analysis of miR-30b expression levels in A549 **(g)** and H1299 **(h)** cells pretreated with miR-30b inhibitor (anti-miR-30b) prior to radiation; levels were normalized to U6. **i** RT-qPCR analysis of *FBXL14* expression levels in A549 cells pretreated with miR-30b inhibitor (anti-miR-30b) prior to radiation; levels were normalized to *ACTB*. **j-l** RT-qPCR analysis of miR-30b expression levels in HCC95 **(j)**, SNU1330 **(k)** cells, and A549 xenograft tumors **(l)** after radiation. **m** Western blot analysis of TWIST1 in NSCLC cells pretreated with anti-miR-30b prior to radiation. **n** Western blot analysis for measurement of the half-life of TWIST1 in A549 cells pretreated with anti-miR-30b prior to radiation, following treatment with CHK as indicated. **o** Representative images and quantification of *in situ* PLA showing the interaction between TWIST1 and FBXL14 in NSCLC cells pretreated with anti-miR-30b prior to radiation. Scale bar = 100 μm.

Data are presented as mean ± SD and analyzed by Student’s t-tests. **p* < 0.05; ***p* < 0.01; ****p* < 0.001


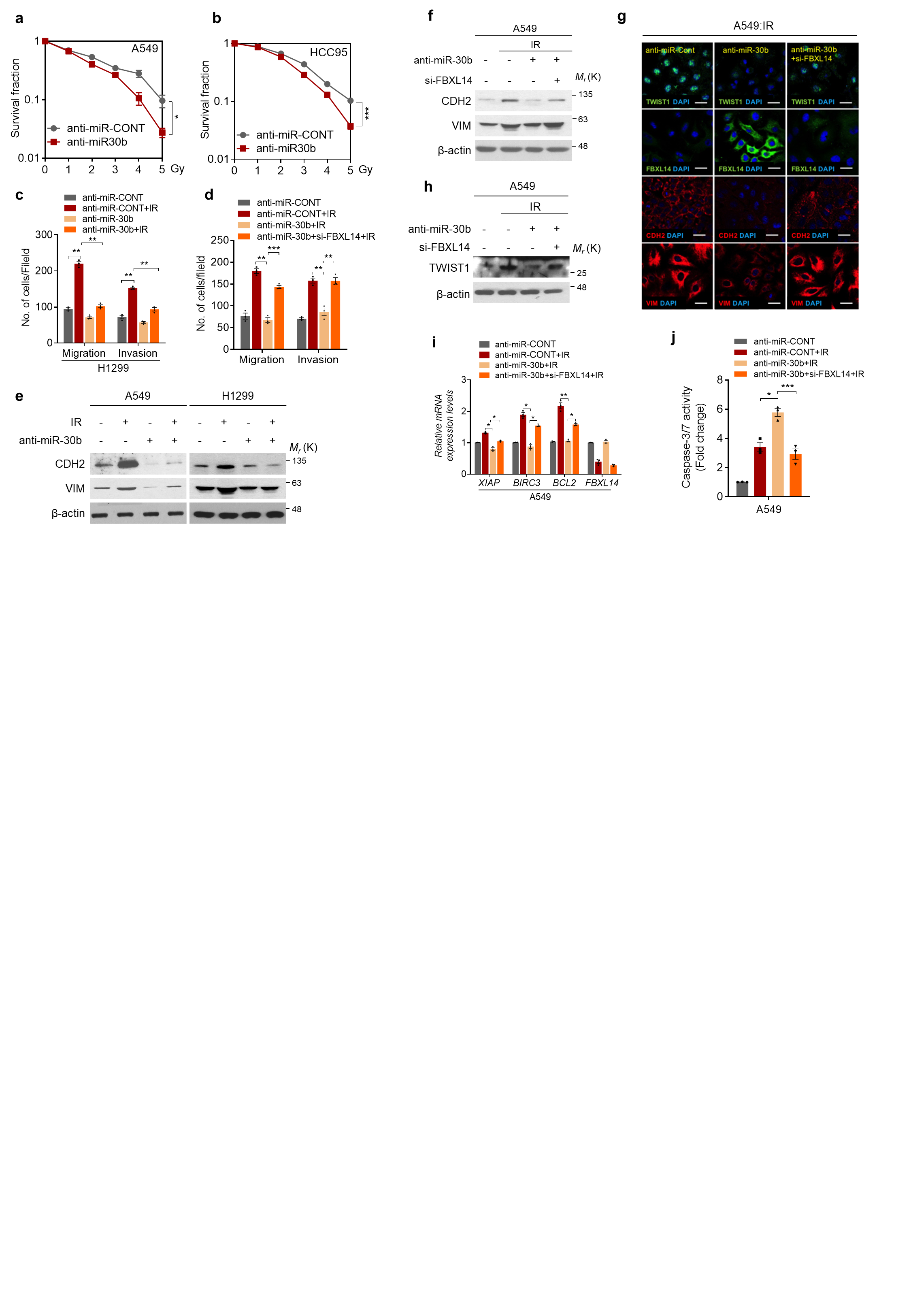


**Supplementary Fig. 8 Radiation-induced EMT in NSCLCs is regulated by miR-30b/FBXL14/TWIST1.** **a-b** Clonogenic survival assay of A549 **(a)** and HCC95 **(b)** cells transfected with anti-miR30b prior to radiation as indicated (n = 3 per group). **c** Transwell migration and invasion assay of H1299 cells transfected with anti-miR-30b prior to radiation as indicated. **d** Transwell migration and invasion assays of A549 cells treated with anti-miR-30b or in combination with si-FBXL14 prior to IR (2 Gy/day×3 days). **e** Western blot analysis of CDH2 and VIM in A549 and H1299 cells transfected with anti-miR-30b prior to radiation. **f** Western blot analysis of CDH2 and VIM in A549 cells transfected with anti-miR-30b alone or in combination with si-FBXL14 prior to radiation. **g** ICC analysis of EMT markers in A549 cells pretreated with anti-miR-30b prior to IR (2 Gy/day×3 days). Scale bar = 100 μm. **h** Western blot analysis of TWIST1 in A549 cells transfected with anti-miR30b alone or in combination with si-FBXL14 prior to radiation as indicated. **i** RT-qPCR analysis of *XIAP, BIRC3, BCL2,* and *FBXL14* expression in A549 cells transfected with anti-miR-30b and/or si-FBXL14 prior to IR. **j** FACS analysis of caspase-3/7 activity in A549 cells transfected with anti-miR-30b and/or si-FBXL14 prior to radiation.

Data are presented as mean ± SD and analyzed by Student’s t-tests. **p* < 0.05; ***p* < 0.01; ****p* < 0.001


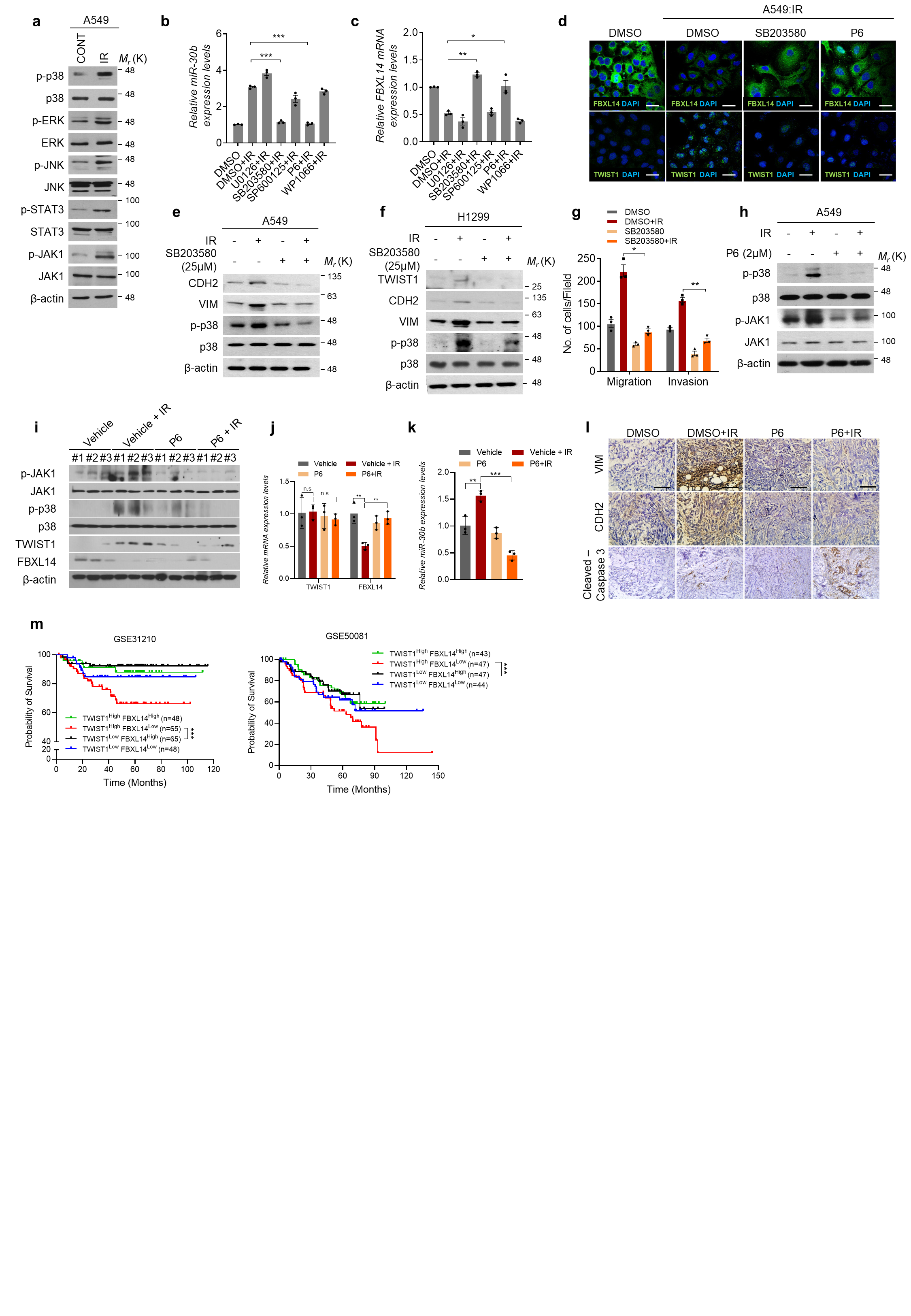


**Supplementary Fig. 9 JAK1/p38 MAPK/miR-30b signaling axis upregulates TWIST1 by negatively regulating FBXL14 in response to radiation.** **a** Western blot analysis of activation of intracellular signaling regulators in A549 cells in response to radiation. **b-c** RT-qPCR analysis of *miR-30b* **(b)** and *FBXL14* **(c)** expression in A549 cells pretreated with U0126 (MEK inhibitor; 10 μM), SB203580 (p38 inhibitor; 25 μM), SP600125 (JNK inhibitor; 10 μM), P6 (JAK inhibitor; 2 μM), WP1066 (STAT3 inhibitor; 2 μM) as indicated. **d** ICC analysis of FBXL14 and TWIST1 in A549 cells pretreated with SB203580 or P6 prior to radiation as indicated. Scale bar = 100 μm. **e-f** Western blot analysis of EMT markers in A549 **(e)** and H1299 **(f)** cells pretreated with SB203580 prior to radiation as indicated. **g** Transwell migration and invasion assay in A549 cells pretreated with SB203580 prior to radiation as indicated. **h** Western blot analysis of p-p38 and p-JAK1 in A549 cells pretreated with P6 prior to IR. **i-l** Western blot **(i),** RT-qPCR **(j, k)**, and IHC analysis **(l)** of xenograft tumors, in which tumors were treated with radiation and/or P6 as indicated. Scale bar = 100 μm (n = 3 mice/group). **m** Kaplan-Meier survival analysis of NSCLC patients (GSE31210, GSE50081); *TWIST1^High^FBXL14^High^*, *TWIST1^High^FBXL14^Low^*, *TWIST1^Low^FBXL14^High^*, *TWIST1^Low^FBXL14^Low^* .

Data are presented as mean ± SD and analyzed by Student’s t-tests. **p* < 0.05; ***p* < 0.01; ****p* < 0.001

**REFERENCES**

1. Koh PK, Faivre-Finn C, Blackhall FH, De Ruysscher D. Targeted agents in non-small cell lung cancer (NSCLC): clinical developments and rationale for the combination with thoracic radiotherapy. *Cancer Treat Rev* **38**, 626-640 (2012).

2. Bussink J, van der Kogel AJ, Kaanders JH. Activation of the PI3-K/AKT pathway and implications for radioresistance mechanisms in head and neck cancer. *Lancet Oncol* **9**, 288-296 (2008).

3. Fang X*, et al.* Deubiquitinase USP13 maintains glioblastoma stem cells by antagonizing FBXL14-mediated Myc ubiquitination. *J Exp Med* **214**, 245-267 (2017).

4. Cui YH*, et al.* FBXL14 abolishes breast cancer progression by targeting CDCP1 for proteasomal degradation. *Oncogene* **37**, 5794-5809 (2018).

5. Brabletz T, Kalluri R, Nieto MA, Weinberg RA. EMT in cancer. *Nat Rev Cancer* **18**, 128-134 (2018).

6. De Craene B, Berx G. Regulatory networks defining EMT during cancer initiation and progression. *Nat Rev Cancer* **13**, 97-110 (2013).

7. Christiansen JJ, Rajasekaran AK. Reassessing epithelial to mesenchymal transition as a prerequisite for carcinoma invasion and metastasis. *Cancer Res* **66**, 8319-8326 (2006).

8. Singh A, Settleman J. EMT, cancer stem cells and drug resistance: an emerging axis of evil in the war on cancer. *Oncogene* **29**, 4741-4751 (2010).

9. Mani SA*, et al.* The epithelial-mesenchymal transition generates cells with properties of stem cells. *Cell* **133**, 704-715 (2008).

10. Tran HD, Luitel K, Kim M, Zhang K, Longmore GD, Tran DD. Transient SNAIL1 expression is necessary for metastatic competence in breast cancer. *Cancer Res* **74**, 6330-6340 (2014).

11. Zheng X*, et al.* Epithelial-to-mesenchymal transition is dispensable for metastasis but induces chemoresistance in pancreatic cancer. *Nature* **527**, 525-530 (2015).

12. Zhang P*, et al.* ATM-mediated stabilization of ZEB1 promotes DNA damage response and radioresistance through CHK1. *Nat Cell Biol* **16**, 864-875 (2014).

13. Voutsadakis IA. Ubiquitination and the Ubiquitin-Proteasome System as regulators of transcription and transcription factors in epithelial mesenchymal transition of cancer. *Tumour Biol* **33**, 897-910 (2012).

14. Zhong J, Ogura K, Wang Z, Inuzuka H. Degradation of the transcription factor Twist, an oncoprotein that promotes cancer metastasis. *Discov Med* **15**, 7-15 (2013).

15. Vernon AE, LaBonne C. Slug stability is dynamically regulated during neural crest development by the F-box protein Ppa. *Development* **133**, 3359-3370 (2006).

16. Lander R, Nordin K, LaBonne C. The F-box protein Ppa is a common regulator of core EMT factors Twist, Snail, Slug, and Sip1. *J Cell Biol* **194**, 17-25 (2011).

17. Lv T*, et al.* Twist1-mediated 4E-BP1 regulation through mTOR in non-small cell lung cancer. *Oncotarget* **6**, 33006-33018 (2015).

18. Avila-Moreno F*, et al.* Overexpression of MEOX2 and TWIST1 is associated with H3K27me3 levels and determines lung cancer chemoresistance and prognosis. *PLoS One* **9**, e114104 (2014).

19. Xu Y*, et al.* Twist1 promotes breast cancer invasion and metastasis by silencing Foxa1 expression. *Oncogene* **36**, 1157-1166 (2017).

20. da Silva SD*, et al.* TWIST1 is a molecular marker for a poor prognosis in oral cancer and represents a potential therapeutic target. *Cancer* **120**, 352-362 (2014).

21. Yi JM*, et al.* Novel methylation biomarker panel for the early detection of pancreatic cancer. *Clin Cancer Res* **19**, 6544-6555 (2013).

22. Liu R, Zheng HQ, Zhou Z, Dong JT, Chen C. KLF5 promotes breast cell survival partially through fibroblast growth factor-binding protein 1-pERK-mediated dual specificity MKP-1 protein phosphorylation and stabilization. *J Biol Chem* **284**, 16791-16798 (2009).

23. Bae S*, et al.* Low-dose gamma-irradiation induces dual radio-adaptive responses depending on the post-irradiation time by altering microRNA expression profiles in normal human dermal fibroblasts. *Int J Mol Med* **35**, 227-237 (2015).

24. Gyorffy B, Surowiak P, Budczies J, Lanczky A. Online survival analysis software to assess the prognostic value of biomarkers using transcriptomic data in non-small-cell lung cancer. *PLoS One* **8**, e82241 (2013).
